# Assessment of the Effectiveness of Pelvic Floor Muscle Training (PFMT) and Extracorporeal Magnetic Innervation (ExMI) in Treatment of Stress Urinary Incontinence in Women: A Randomized Controlled Trial

**DOI:** 10.1155/2020/1019872

**Published:** 2020-01-16

**Authors:** Magdalena Weber-Rajek, Agnieszka Strączyńska, Katarzyna Strojek, Zuzanna Piekorz, Beata Pilarska, Marta Podhorecka, Kinga Sobieralska-Michalak, Aleksander Goch, Agnieszka Radzimińska

**Affiliations:** ^1^Department of Physiotherapy, Collegium Medicum in Bydgoszcz, Nicolaus Copernicus University, Torun, Poland; ^2^Clinic of Urology, Collegium Medicum in Bydgoszcz, Nicolaus Copernicus University, Torun, Poland; ^3^Department of Geriatrics, Collegium Medicum in Bydgoszcz, Nicolaus Copernicus University, Torun, Poland; ^4^Clinic of Rehabilitation, 10th Military Research Hospital and Polyclinic, Bydgoszcz, Poland

## Abstract

**Objective:**

The purpose of this study is to assess the effectiveness of pelvic floor muscle training and extracorporeal magnetic innervation in treatment of urinary incontinence in women with stress urinary incontinence.

**Methods:**

The randomized controlled trial enrolled 128 women with stress urinary incontinence who were randomly allocated to either one out of two experimental groups (EG1 or EG2) or the control group (CG). Subjects in the experimental group 1 (EG1) received 12 sessions of pelvic floor muscle training, whereas subjects in the experimental group 2 (EG2) received 12 sessions of extracorporeal magnetic innervation. Subjects in the control group (CG) did not receive any therapeutic intervention. The following instruments were used to measure results in all study groups at the initial and final assessments: Revised Urinary Incontinence Scale (RUIS), Beck Depression Inventory (BDI-II), General Self-Efficacy Scale (GSES), and King's Health Questionnaire (KHQ).

**Results:**

In both experimental groups, a statistically significant decline in depressive symptoms (BDI-II) and an improvement in urinary incontinence severity (RUIS) and quality of life (KHQ) were found in the following domains: “social limitations,” “emotions,” “severity measures,” and “symptom severity scale.” Moreover, self-efficacy beliefs (GSES) improved in the experimental group that received ExMI (EG2). No statistically significant differences were found between all measured variables in the control group. Comparative analysis of the three study groups showed statistically significant differences at the final assessment in the quality of life in the following domains: “physical limitations,” “social limitations,” “personal relationships,” and “emotions.” *Conclusion*. Pelvic floor muscle training and extracorporeal magnetic innervation proved to be effective treatment methods for stress urinary incontinence in women. The authors observed an improvement in both the physical and psychosocial aspects.

## 1. Introduction

The World Health Organization (WHO) and the International Continence Society (ICS) define urinary incontinence (UI) as an involuntary leakage of urine through the urethra and consider it a health, social, and hygienic concern [1].

The Standardisation Steering Committee (SSC) recognizes three main types of UI: stress urinary incontinence (SUI), urge urinary incontinence (UUI), and mixed urinary incontinence (MUI). While SUI is the most common type of UI, there is inconclusive data on the frequency of urinary incontinence in the general population. During the Global Forum on Incontinence, held on April 17-18th, 2018, in Rome, it was assumed that UI affects 6–10% of the general population [2]. It is worth mentioning that, according to the WHO data, any condition affecting at least 5% of the population is recognized as a social disease. The above-mentioned data confirm the gravity of urinary incontinence and the importance of disease prevention and treatment. Nowadays, increasing attention is given to physiotherapy as a conservative treatment for urinary incontinence. The most important physiotherapy treatments for UI include pelvic floor muscle training, electrostimulation, biofeedback, and magnetotherapy. In the following series of studies, the authors assessed the effectiveness of pelvic floor muscle training (PFMT) and extracorporeal magnetic innervation (ExMI). The European Association of Urology (EAU) recommends the use of pelvic floor muscle training as a basic nonsurgical treatment for UI [3], whereas extracorporeal magnetic innervation (ExMI) is a rather new physiotherapy method used in the treatment of urinary incontinence. ExMI uses high electromagnetic induction values (2 Tesla) with a frequency of 10–50 Hz, which is adjusted depending on the type of urinary incontinence. During an ExMI treatment session, patients are seated in a special chair with a magnetic field generator in the seat. The magnetic field emitted by the generator penetrates pelvis minor organs and acts on motor fibers of pudendal and visceral nerves. Once the sodium-potassium pump is activated and the motor neuron depolarization begins, nerve impulses reach the neuromuscular junction which consequently initiates muscle contraction [4–6]. Nevertheless, there are relatively few scientific reports that assess the effectiveness of ExMI. The EAU pointed this out as well in their 2017 guidelines on the nonsurgical treatment for urinary incontinence [3].

Urinary incontinence is a multifaceted issue that impairs patients' physical and psychosocial functioning. In light of the above, improving patients' general quality of life, that is, physical, mental, and social well-being, should be paramount in the treatment of urinary incontinence.

### 1.1. Study Purpose

This study aims to compare the effectiveness of pelvic floor muscle training and extracorporeal magnetic innervation in the treatment of stress urinary incontinence.

## 2. Methods

### 2.1. Study Design

**Figure 1:**
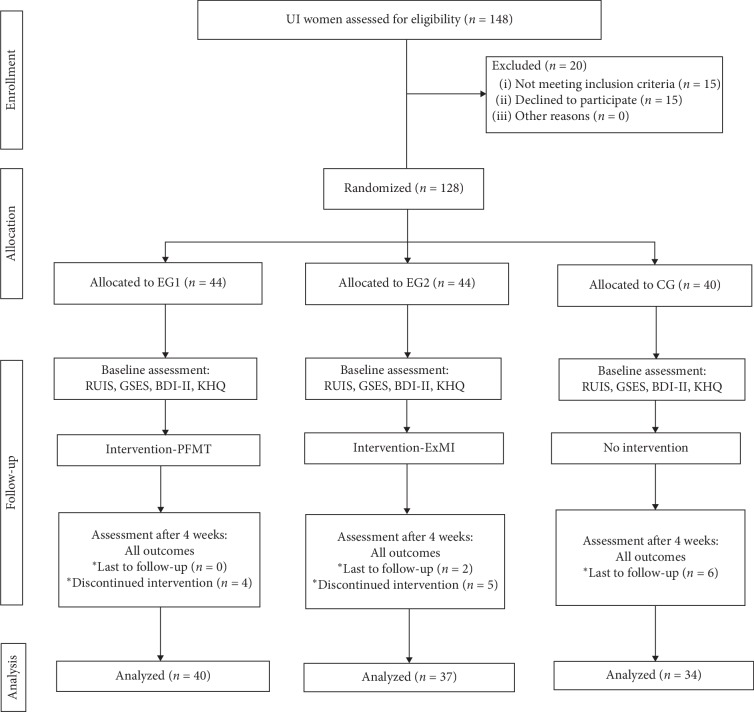
The study flow diagram. RUIS: Revised Urinary Incontinence Scale; GSES: General Self-Efficacy Scale; BDI-II: Beck Depression Inventory; KHQ: King's Health Questionnaire; UI: urinary incontinence.; PFMT: pelvic floor muscle training; ExMI: extracorporeal magnetic innervation.

In the period between February 2017 and June 2018, 148 participants affected by urinary incontinence were enrolled in a randomized controlled trial. The study was conducted in accordance with the Declaration of Helsinki guidelines and with the approval of a local Bioethics Committee. Moreover, all participants provided a statement confirming that written informed consent was given. All the deidentified data is presented in the report. The authors stratified randomization by allocating participants to one of the three study groups. The allocation method was simple—each subject picked a sealed envelope with a computer-generated group allocation number. Furthermore, the main investigator was blinded to the study group allocation. Among the 20 subjects excluded from the study, 15 subjects failed to meet the inclusion criteria, and 5 subjects refused to participate. 128 subjects who met the inclusion criteria were then randomly allocated to one out of the two experimental groups (EG1 or EG2) or the control group (CG). Of the 17 women who failed to complete the study, 9 subjects withdrew from EG1 and EG2 during the 4-week treatment program, 6 subjects from the CG missed the final study visit, and 2 subjects from the EG2 submitted incomplete questionnaires. Consequently, 111 women aged 45 to 78 (mean 68.77) successfully completed the study (PFMT (EG1) was *n* = 40 aged 60 to 78 (mean 70.12), ExMI (EG2) was *n* = 37 aged 45 to 76 (mean 66.71), and CG was *n* = 34 aged 60 to 78 (mean 69.79)). Applying the CONSORT statement (Consolidated Standards of Reporting Trials) (Figure 1) allowed the authors to improve the RCT reporting quality [7]. Prior to the treatment, subjects provided information on the contraindications to the treatment, circumstances of urine loss, and any comorbid conditions. It should be noted that the type of urinary incontinence was diagnosed by a urology specialist. Study inclusion criteria were as follows: diagnosed SUI, and no contradictions to the PFMT or ExMI treatment.

Contradictions to PFMT were as follows: active malignancy, recent surgeries, recent pelvic fractures, fever, acute inflammations, uterine tumors and myomas, urinary or genital tract infections, grade 3 or 4 hemorrhoids, stage 3 uterine prolapse (downward displacement of the uterus into the vagina) [8].

Contradictions to ExMI were as follows: pregnancy, recent pelvic fractures, fever, acute inflammations, active malignancy, uterine tumors and myomas, stage 3 uterine prolapse, hemorrhoids, urinary or genital tract infections, suspected urethral and/or vesical fistula, severe urethral sphincter weakness and/or defect, deep vein thrombosis, acute infections, cardiac arrhythmia, cardiac pacemaker, and neurological diseases [6].

Study exclusion criteria were as follows: presence of contraindications to the PFMT or ExMI treatment, diagnosed MUI or UUI, and recent therapeutic interventions in UI 3 months prior to the study (PFMT, ExMI, electrostimulation, or biofeedback).

### 2.2. Measurements

#### 2.2.1. The Revised Urinary Incontinence Scale (RUIS)

The RUIS is a valid 5-item scale used to determine UI symptoms and to monitor treatment outcomes. A score of less than 3 indicates no urinary incontinence; a score of 4–8 is considered mild urinary incontinence; a score of 9–12 is considered moderate urinary incontinence; a score of 13 or above indicates severe urinary incontinence [9].

#### 2.2.2. The General Self-Efficacy Scale (GSES)

The scale was developed by Matthias Jerusalem and Ralf Schwarzer to assess merely one psychometric characteristic—one's general self-efficacy belief, which is understood as a belief in one's capabilities to deal with various situations. The obtained raw scores were afterwards transformed into standardised sten scores, and higher scores indicate higher self-efficacy beliefs. Score ranges are defined as follows: sten scores of 1–4 indicate low scores; sten scores of 5–6 indicate average scores; sten scores of 7–10 indicate high scores [10].

#### 2.2.3. Beck Depression Inventory-II (BDI-II)

The BDI-II questionnaire is a self-report depression scale widely used in research on mental disorders, and it is still as popular as ever. The tool allows to evaluate the mood of urological, gynecological, oncological, and neurological patients. The questionnaire is a 21-item scale with individual item scores ranging from 0 (no symptoms) to 3 (severe symptoms). Score ranges are defined as follows: a score of 0–8 indicates no depression, and a score of 9–18 indicates moderate depression, whereas a score of 18 or above indicates severe depression [11].

#### 2.2.4. King's Health Questionnaire (KHQ)

The KHQ is a 21-item patient self-report scale that comprises 3 parts [12, 13].

Part 1 (KHQ–1) focuses on the general health perception (one item) and the incontinence impact (one item).

Part 2 (KHQ–2) focuses on the following:

- Role limitations (two items)–2A
- Physical limitations (two items)–2B
- Social limitations (two items)–2C
- Personal relationships (three items)–2D
- Emotions (three items)–2E
- Sleep/energy (two items)–2F
- Severity measures (four items)–2G

Part 3 (KHQ–3) is a single-item section that contains ten bladder-related symptoms—frequency, nocturia, urgency, urge, stress, intercourse incontinence, nocturnal enuresis, infections, pain, and difficulty in voiding—which are answered on a 4-point scale. The subscales in parts 1 and 2 are scored from 0 (the best quality of life) to 100 points (the worst quality of life), whereas the scale in part 3 is scored from 0 (the best quality of life) to 30 points (the worst quality of life). The lower the KHQ score, the better the quality of life.

### 2.3. Intervention

#### 2.3.1. Pelvic Floor Muscle Training

Women in the EG1 received 12 PFMT therapy sessions (45-minute sessions, 3 times a week, for 4 weeks) that followed a specific training regimen. The exercises were supervised by a physiotherapist and completed in five/six-person groups. Prior to the training session, study participants were examined for posture and body correction, learned how to mobilize sacroiliac joints, and improve the lumbar-sacral spine, as well as hip and knee joint range of movement. The subjects also participated in abdominal breathing exercises simulating the thoracic duct. The pelvic floor muscle training (PMFT) regimen focused on straining fast- and slow-twitch muscle fibers of the pelvic floor using the transversus abdominis muscle tension technique. The exercises were performed with and without changing the position, with relaxed gluteal muscles, and the muscle function was synchronized with breathing. The PFMT exercises were performed in standing, sitting, and lying positions. The number of exercises and repetitions were individually determined and matched to the subjects' functional abilities [8].

#### 2.3.2. Extracorporeal Magnetic Innervation

Women in the EG2 received 12 ExMI therapy sessions (15-minute sessions, 3 times a week, for 4 weeks) which were delivered using the NeoControl chair (Neotonus Inc., Marietta, GA, USA). The applied magnetic field parameters were as follows: 2.0 Tesla at 50 Hz, delivered for 8 seconds with a dwell time of 4 seconds. During consecutive treatment sessions, the field intensity was being increased from 20% to 100%, and the electromagnetic stimulation strength corresponded to the highest level tolerated by the patient [6].

### 2.4. Statistical Analyses

The authors analyzed the collected data using the PQ Stat 1.6.8 software. Also, the Shapiro–Wilk test was implemented to check the normality distribution of measured variables. The results in the initial and final assessments in the study groups were compared using the Wilcoxon test. The Anova Kruskal–Wallis test was applied to determine the differences between the three study groups. Then, the authors performed a post hoc Conover-Iman test. The statistical significance level was defined as *P* < 0.05.

## 3. Results

Table 1 presents Wilcoxon test results and descriptive statistics for all measured variables across both experimental groups and the control group at the initial and final assessments.

Table 2 presents the Anova Kruskal–Wallis test applied to determine the differences between the three study groups at the final assessment.

A statistically significant difference was found in the quality of life results obtained from the three study groups in the following domains: physical limitations (KHQ–2B), social limitations (KHQ–2C), personal relationships (KHQ–2D), and emotions (KHQ–2E).

In the next stage of the study, the authors performed a post hoc Conover-Iman test. This is illustrated by the results in Table 3.

Post hoc Conover-Iman test results showed no statistically significant differences between experimental groups and showed statistically significant differences between the experimental groups and the control group in all analyzed variables.

## 4. Discussion

The authors assessed the physical and psychosocial functioning of women with stress urinary incontinence following different physiotherapy treatment methods for UI.

The physical aspects were assessed using the Revised Urinary Incontinence Scale (RUIS), which is a reliable and valid instrument used to determine urinary incontinence symptoms and monitor patient response to treatment [9]. The RUIS scores showed a statistically significant improvement in the urinary incontinence severity in both the PFMT group (EG1) and the ExMI group (EG2). Similar results were obtained in our previous studies [6, 8, 14].

“Severity measures” domain in Part 2 of the King's Health Questionnaire (KHQ–2G) was also used to assess the incontinence severity. The authors observed a statistically significant improvement in this domain following both PFMT and ExMI.

In our previous studies, we assessed the level of myostatin concentration after using PFMT and ExMI in a group of older women with UI [6, 8]. The level of myostatin increases in the periods of skeletal muscle inactivity, and the inhibition of serum myostatin increases muscle strength and mass. Therapeutic interventions such as physical activity can suppress myostatin signalling ameliorate the effects of advancing age on skeletal muscle mass and function. Some studies suggest that myostatin inhibits human urethral rhabdosphincter satellite cell proliferation; therefore, inhibition of myostatin function might be a useful strategy for the treatment of stress UI. The results of these studies showed that effective PFMT and ExMI cause downregulation of myostatin concentration and an improvement in the severity of urinary incontinence in elderly women with stress UI [6, 8].

International Continence Society guidelines acknowledged in their recommendations that comprehensive patient care needs to take into consideration physical and psychosocial perspectives [15]. Other parameters measured during this study were self-efficacy beliefs (GSES), depression symptoms (BDI-II), and quality of life (KHQ).

General self-efficacy is an essential mental resource that may influence behavioral determinants and consequently impact, either directly or indirectly, health-related behaviors. People with high self-efficacy beliefs are more likely to practice health-related behaviors because they strongly believe in their ability to overcome obstacles and achieve their goals [16]. The analyses of self-efficacy beliefs among UI patients may provide essential information on self-motivation and belief in planned intervention effectiveness [17, 18]. The study results demonstrated that there was a statistically significant increase in self-efficacy beliefs in the ExMI group (EG2), while no changes in self-efficacy beliefs were observed in the PFMT group (EG1). More importantly, relatively high self-efficacy beliefs were recorded in both experimental groups before the treatment. Therefore, it can be assumed that high self-efficacy beliefs motivated the patients to face the problem of urinary incontinence and participate in our study. We have also assessed the self-efficacy after using ExMI in women with UI in previous studies, which led to similar conclusions [6].

Among comorbid conditions associated with urinary incontinence and frequently reported in the literature on the subject, depression emerges as the most debilitating mental health condition [19–22]. The Beck Depression Inventory administered to the PFMT group at the initial assessment yielded the following results: no depression in 22 patients (55%), moderate depression in 14 patients (35%), and severe depression in 4 patients (10%), whereas the following results were reported after the treatment: no depression in 29 patients (73%), moderate depression in 9 patients (22%), and severe depression in 2 patients (5%).

The Beck Depression Inventory administered to the ExMI group at the initial assessment yielded the following results: no depression in 20 patients (54%), moderate depression in 14 patients (38%), and severe depression in 3 patients (8%), whereas the following results were reported after the treatment: no depression in 27 patients (73%), moderate depression in 8 patients (22%), and severe depression in 2 patients (5%). We observed a statistically significant decline in depressive symptoms in both experimental groups after the treatment.

Moreover, the King's Health Questionnaire was used to evaluate patients' quality of life. The tool was designed in 1997 by Dr. C. J. Kelleher and his colleagues from the Department of Urogynaecology at King's College, Cambridge. Once the questionnaire's reliability and validity were confirmed with standard psychometric techniques during six pilot studies, the final version of the tool was published in the British Journal of Obstetrics and Gynaecology in December 1997 [12]. The KHQ was proved to be an essential and reliable tool for the evaluation of the quality of life in UI female patients, and the instrument is also recommended by the European Clinical Practice Guidelines [13].

In this study, a statistically significant improvement in the quality of life was reported in both experimental groups in the following domains: “social limitations” (KHQ–2C), “emotions” (KHQ–2E), “severity measures” (KHQ–2G), and “symptom severity scale” (KHQ–3).

No statistically significant differences emerged between all measured variables in the control group. Comparative analysis of the three study groups showed statistically significant differences in the quality of life at the final assessment in the following domains: “physical limitations” (KHQ–B), “social limitations” (KHQ–C), “personal relationships”(KHQ–2D), and “emotions” (KHQ–2E). Additionally, post hoc Conover-Iman test results showed no statistically significant differences between experimental groups and showed statistically significant differences between the experimental groups and the control group in all analyzed variables.

The study results demonstrated that both physiotherapy treatment methods are effective in the treatment of urinary incontinence. The authors observed an improvement in both the physical and psychosocial parameters. However, the authors want to highlight that there are more contradictions to the ExMI therapy than to PFMT. It is worth mentioning that two subjects during our study withdrew from the ExMI therapy due to the discomfort experienced during the treatment.

## 5. Conclusions

1. Pelvic floor muscle training and extracorporeal magnetic innervation proved to be effective treatment methods for stress urinary incontinence in women. The authors observed an improvement in both the physical and psychosocial aspects.
2. In both experimental groups, there was a statistically significant decline in depressive symptoms, and there was an improvement in urinary incontinence severity and quality of life in the following domains: “social limitations,” “emotions,” “severity measures,” and “symptom severity scale,” following the treatment.
3. Moreover, higher self-efficacy beliefs were observed in the experimental group that received ExMI.
4. Comparative analysis of the three study groups showed statistically significant differences in the quality of life at the final assessment in the following domains: “physical limitations,” “social limitations,” “personal relationships,” and “emotions.”

## Figures and Tables

**Table 1:** Comparative analysis of all measured variables for the EG1, EG2, and CG at the initial and final assessments.

		EG1 (*n* = 40)			EG2 (*n* = 37)			CG (*n* = 34)		
Parameter	Statistics	Initial assessment	Final assessment	*P* value	Initial assessment	Final assessment	*P* value	Initial assessment	Final assessment	*P* value
RUIS	Med	8.00	6.00	<0.001^*∗*^	9.00	7.00	0.001^*∗*^	8.00	9.00	0.190
	IQR	6.00	5.00		6.00	4.00		7.00	5.00	
GSES	Med	7.00	7.00	0.231	7.00	7.00	0.019^*∗*^	7.00	7.00	0.147
	IQR	4.00	3.00		3.00	3.00		3.00	1.00	
BDI-II	Med	6.00	5.00	<0.001^*∗*^	7.00	5.00	<0.001^*∗*^	6.50	7.50	0.856
	IQR	9.00	7.50		11.00	7.00		11.00	9.00	
KHQ–1	Med	16.66	16.66	0.146	16.66	33.33	0.113	16.66	33.33	8.817
	IQR	50.00	33.33		50.00	33.33		50.00	50.00	
KHQ–2A	Med	33.33	24.99	0.073	33.33	33.33	0.463	33.33	33.33	0.932
	IQR	33.33	50.00		33.33	50.00		33.33	40.97	
KHQ–2B	Med	5.55	0.00	0.089	33.33	0.00	0.322	11.11	22.22	0.297
	IQR	33.33	22.22		0.00	22.22		44.44	48.61	
KHQ–2C	Med	33.33	11.11	0.001^*∗*^	33.33	22.22	0.001^*∗*^	33.33	22.22	0.536
	IQR	50.00	22.22		50.00	22.22		35.41	31.94	
KHQ–2D	Med	22.22	16.66	0.444	22.22	33.33	0.327	22.22	44.44	0.255
	IQR	13.88	41.66		22.22	50.00		33.33	45.83	
KHQ–2E	Med	33.33	16.66	0.035^*∗*^	41.66	33.33	0.004^*∗*^	37.50	50.00	0.789
	IQR	41.66	50.00		50.00	50.00		47.91	31.25	
KHQ–2F	IQR	50.00	41.66	0.699	33.33	41.66	0.405	29.16	38.19	0.663
	Med	33.33	33.33		33.33	41.66		33.33	41.66	
KHQ–2G	IQR	41.66	33.33	<0.001^*∗*^	41.66	25.00	<0.001^*∗*^	41.66	31.25	0.190
	Med	41.66	16.66		33.33	16.66		33.33	33.33	
KHQ–3	IQR	11.25	7.00	<0.001^*∗*^	6.00	6.00	<0.001^*∗*^	5.00	7.50	0.609
	Med	6.00	2.00		6.00	2.00		6.75	4.50	

^*∗*^Statistical significance. EG1: experimental group 1; EG2: experimental group 2; CG: control group; Med: median; IQR: interquartile range; *P*: significance level.

**Table 2:** Comparison of all measured variables across the three study groups at the final assessment.

Parameter	Group	H statistics	*P* value
RUIS	EG1	5.066	0.079
	EG2		
	CG		

GSES	EG1	4.120	0.127
	EG2		
	CG		

BDI-II	EG1	0.166	0.920
	EG2		
	CG		

KHQ–1	EG1	1.479	0.473
	EG2		
	CG		

KHQ–2A	EG1	1.318	0.517
	EG2		
	CG		

KHQ–2B	EG1	8.211	0.016^*∗*^
	EG2		
	CG		

KHQ–2C	EG1	7.785	0.020^*∗*^
	EG2		
	CG		

KHQ–2D	EG1	7.762	0.020^*∗*^
	EG2		
	CG		

KHQ–2E	EG1	9.046	0.010^*∗*^
	EG2		
	CG		

KHQ–2F	EG1	1.331	0.513
	EG2		
	CG		

KHQ–2G	EG1	10.457	0.066
	EG2		
	CG		

KHQ–3	EG1	3.957	0.138
	EG2		
	CG		

^*∗*^Statistical significance.

**Table 3:** Post hoc Conover-Iman test.

		EG1	EG2	CG
*KHQ–2B*				
Statistics	EG1		0.192	2.670
	EG2	0.192		2.440
	CG	2.670	2.440	
*P* value	EG1		0.847	0.008^*∗*^
	EG2	0.847		0.016^*∗*^
	CG	0.008^*∗*^	0.016^*∗*^	

*KHQ–2C*				
Statistics	EG1		0.430	2.694
	EG2	0.430		2.237
	CG	2.694	2.237	
*P* value	EG1		0.667	0.008^*∗*^
	EG2	0.667		0.027^*∗*^
	CG	0.008^*∗*^	0.027^*∗*^	

*KHQ–2D*				
Statistics	EG1		0.547	2.737
	EG2	0.547		2.155
	CG	2.737	2.155	
*P* value	EG1		0.584	0.007^*∗*^
	EG2	0.584		0.033^*∗*^
	CG	0..007^*∗*^	0.033^*∗*^	

*KHQ–2E*				
Statistics	EG1		0.014	2.715
	EG2	0.014		2.689
	CG	2.715	2.689	
*P* value	EG1		0.988	0.007^*∗*^
	EG2	0.988		0.008^*∗*^
	CG	0.007^*∗*^	0.008^*∗*^	

^*∗*^Statistical significance.

## Data Availability

The data used to support the findings of this study are available from the corresponding author upon request.

## Additional Points

*Limitation.* The authors acknowledge certain limitations of their analysis. These include a relatively small study group and the lack of assessment of long-term treatment outcomes.
